# Association Between c-Myc and Colorectal Cancer Prognosis: A Meta-Analysis

**DOI:** 10.3389/fphys.2018.01549

**Published:** 2018-11-13

**Authors:** Wei-Ling He, Xiang-Tao Weng, Jue-Lian Wang, Yong-Kai Lin, Tian-Wen Liu, Qian-Yi Zhou, Yue Hu, Yunbao Pan, Xin-Lin Chen

**Affiliations:** ^1^Department of Gastrointestinal Surgery, The First Affiliated Hospital, Sun Yat-sen University, Guangzhou, China; ^2^The Second Clinical College, Guangdong Provincial Hospital of Chinese Medicine, Guangzhou University of Chinese Medicine, Guangzhou, China; ^3^The First Clinical College, Guangzhou University of Chinese Medicine, Guangzhou, China; ^4^School of Basic Medical Science, Guangzhou University of Chinese Medicine, Guangzhou, China; ^5^Department of Laboratory Medicine, Zhongnan Hospital of Wuhan University, Wuhan University, Wuhan, China

**Keywords:** c-Myc, colorectal cancer, prognosis, biomarker, meta-analysis

## Abstract

**Background:** There is debate as to whether c-Myc predicts prognosis in colorectal cancer (CRC). In this study, we aimed to review the association between c-Myc and CRC prognosis.

**Methods:** Pertinent studies were identified by searching electronic databases and carefully reviewing the reference lists of pertinent studies until March 2016. The summary hazard ratio (HR) and corresponding 95% confidence interval (CI) were calculated to study the association between c-Myc and CRC prognosis.

**Results:** Eight cohort studies (including seven studies about overall survival [OS] and one study about disease free survival [DFS]) were included. The pooled HR of OS was 1.13 (95% CI: 0.66–1.95). In subgroup analysis, no significant association between c-Myc and CRC prognosis was found in the studies either from Western countries (HR: 0.87, 95% CI: 0.68–1.10) or Asian countries (HR: 1.89, 95% CI: 0.62–5.77). HRs were 0.86 (95% CI: 0.38–1.94) and 1.57 (95% CI: 0.73–3.39) for the studies using univariate analysis and multivariate analysis, respectively. HR from the studies that examined DNA level was significantly different (HR: 2.05, 95% CI: 1.22–3.46); while that about RNA level or protein level was not significantly different.

**Conclusion:** c-Myc was not associated with CRC prognosis in this meta-analysis. However, the conclusion is preliminary and should be examined in future studies.

## Introduction

Colorectal cancer (CRC) is the third most frequent cancer worldwide and the fourth most common cause of cancer-related death (Torre et al., 2015). There are approximately 1.4 million new cases and 700,000 deaths from CRC each year (Torre et al., 2015). CRC is a heterogeneous and complicated disease affected by both environmental and genetic factors. A number of cancer-related genes are correlated with CRC prognosis, but the survival benefit associated with targeted therapies is only 4–5 months (Bokemeyer et al., 2012), indicating that the precise molecular mechanisms of CRC are unclear.

The Myc family encodes three highly related nuclear phosphoproteins: c-Myc, l-Myc, and n-Myc (Mukherjee et al., 1992). c-Myc functions as an oncogene, participating in cell growth, death, transformation, and therapy sensitivity (Hermeking and Eick, 1994; Lee K.B. et al., 2016; Wang et al., 2016). The c-Myc protein occupies regulatory regions of up to 15% of all genes and can both activate or suppress various target genes (Dang et al., 2006; Feng et al., 2016). The target genes of c-Myc are involved in various cellular functions, including survival, cell cycle, protein synthesis, cell adhesion, and non-coding RNA expression (Dang et al., 2006).

Aberrant expression of c-Myc was observed in many human cancers and was elevated in up to 70–80% of CRC (Erisman et al., 1985). Several studies have focused on the association between c-Myc and CRC prognosis. Bhatavdekar et al. (1997) reported that measurement of c-Myc expression in primary CRC tissue did not predict prognosis (Erisman et al., 1988). However, some studies showed that positive c-Myc expression had the strongest association with poor survival in CRC patients (Rowley et al., 1990; Bhatavdekar et al., 1997; Kakisako et al., 1998; Lee et al., 2015). For example, Lee et al. (2015) indicated that c-Myc was an independent factor for poor prognosis in consecutive CRC patients according to multivariate analysis. On the other hand, several studies found that c-Myc was correlated with a favorable prognosis of CRC patients (Smith and Goh, 1996; Bockleman et al., 2012; Toon et al., 2014). For example, Smith and Goh (1996) demonstrated that overexpression of c-Myc mRNA in CRC tumors was associated with a better prognosis. All these findings suggested that the prognostic value of c-Myc in CRC remained controversial and inconclusive. Therefore, we conducted a meta-analysis to evaluate the association between c-Myc and CRC prognosis.

## Materials and Methods

### Literature Search

The Research Ethics Committee of Guangzhou University of Chinese Medicine provided ethical approval. PubMed, EMBASE, ISI Web of Knowledge, and the Cochrane Database were searched for eligible studies up to March 14th, 2016. The search strategy was carried out using the following words: “colorectal” (large intestine, large bowel, colon, colonic, rectal or rectum), “cancer” (carcinoma, tumor, neoplasm or cancers), “c-Myc” and “prognosis” (prognoses, prognostic, predictive, biomarker, marker, survival, survive, cox, log-rank or Kaplan-Meier). The search strategy for the Pubmed database was shown in Appendix 1. The reference lists of pertinent publications were also checked for the eligible studies. Only studies published in English were included. In case of duplicate reports or of studies obviously reporting results from the same study, only the latest published studies were selected. This meta-analysis was performed according to the preferred reporting items for systematic reviews and meta-analysis (PRISMA statement) (Moher et al., 2009). The PRISMA 2009 Checklist was shown in Appendix 2.

### Selection Criteria

The inclusion and exclusion criteria consisted of the following three aspects: (1) studies of colorectal cancer (including colon cancer, or rectal cancer) were included; (2) the relationship between c-Myc and patients’ prognosis [i.e., overall survival (OS), disease free survival (DFS), or relapse free survival (RFS)] was studied; and the hazard ratio (HR) and its 95% confidence interval (CI) were provided; and (3) studies were published in the English language. The eligible studies included cohort studies and randomized control trials.

### Data Extraction

The titles and abstracts of all the studies were screened by two of three reviewers independently (X-tW, J-lW, and Y-kL). The eligible or uncertain studies were retrieved for the full texts. Two of three reviewers (X-tW, J-lW and Y-kL) read the full texts and identified the eligible publications. For each eligible study, the following information was extracted: first author, year of publication, country of origin, study time, study type, sample sizes, the characteristics of the patients (gender, stage, differentiation, and treatment method), median follow-up time, the c-Myc information (proportion of positive c-Myc, test sample, test content, and analytic method) and prognosis. Country of origin was categorized as Western countries and Asian countries. Disagreements in data collection were resolved by consensus.

### Statistical Analysis

The association between c-Myc and CRC survival was examined using HR with its 95% CI. DFS and OS were analyzed separately.

The heterogeneity of the individual HR was calculated using Chi-square tests. A heterogeneity test with inconsistency index statistic (*I*^2^) and *Q* statistic were carried out (Handoll, 2006). The *Q* test suggested lack of heterogeneity when *P* > 0.10, and summary HR was examined using fixed-effect model (Mantel and Haenszel, 1959). Otherwise, random-effect model was executed (DerSimonian and Laird, 1986). Subgroup analysis were conducted according to different countries (West [Europe and America], and Asia), analytic methods (univariate analysis, multivariate analysis) and test content (Protein, DNA, RNA). Meta-regression was performed to find out the factors related with the heterogeneity of the HRs. A sensitivity analysis was carried out to evaluate the stability of the results. In addition, Egger’s test and funnel plots were utilized to evaluate publication bias. All statistical analyses were conducted using STATA software (version 12.0).

## Results

### Characteristics of Studies

**FIGURE 1:**
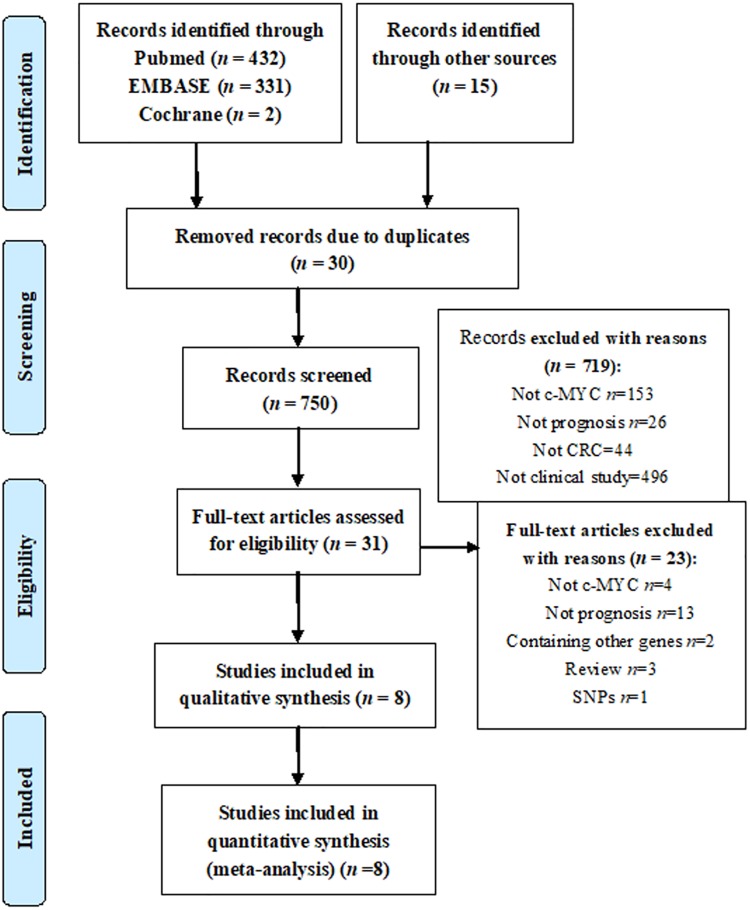
Flow chart of the literature search and study selection.

The initial search strategy identified 780 potentially eligible studies. Thirty studies were excluded because of duplication. We excluded 719 studies after detailed review of the abstract. The remaining 31 studies were evaluated for the full texts. Four studies did not involve c-Myc, thirteen studies did not deal with prognosis, two included other genes, three were review articles, and one was about single-nucleotide polymorphism and was therefore excluded. Eventually, we included eight studies in our meta-analysis (Figure 1; Erisman et al., 1988; Rowley et al., 1990; Smith and Goh, 1996; Bhatavdekar et al., 1997; Kakisako et al., 1998; Bockleman et al., 2012; Toon et al., 2014; Lee et al., 2015).

Three studies were from Asian countries (Smith and Goh, 1996; Bhatavdekar et al., 1997; Kakisako et al., 1998; Lee et al., 2015), and others were from Western countries. A total of 2,947 patients were included (Table 1). All of the eligible studies were cohort studies. The proportion of patients with positive c-Myc was ≥60%, except the study by Bockleman et al. (2012) (Table 2). One study reported DFS, while others reported OS (Table 2). The HR from the only one study about DFS of c-Myc was 5.81 (95% CI: 1.02–32.96; 35 patients). The following results were based on OS.

**Table 1:** The characteristics of included studies.

Author	Country	Year of publication	Study time	Study type	Sample size	Male (%)	Mean of age (range)	Stage (I + II, %)	Differentiation (well +moderate, %)	Surgery (%)	Chemotherapy (%)	Radiotherapy (%)	Median follow-up time (range, month)
Bhatavdekar JM	India	1996	1988–1991	Cohort	48	66.7	48 (25–74)	50.0	81.3	100.0	79.2	41.7	30.0^∗^ (2–36)
Böckelman C	Finland	2012	1989–2001	Cohort	540	55.0	65^∧^ (NR)	50.4	NR	36.3	NR	NR	56.4 (0–296)
Erisman MD	United States	1988	1983–1984	Cohort	38	57.9	70.6^∧^ (55–95)	60.5	42.1	15.8	NR	NR	40.25 (NR)
Kakisako K	Japan	2015	1990–1993	Cohort	35	51.4	65 (51–84)	48.6	94.3	100.0	NR	NR	≥60 (NR)
Lee KS	South Korea	1990	2005–2006	Cohort	367	55.9	64.2 (NR)	44.1	90.2	100.0	NR	NR	55^∗^ (1–85)
Rowley S	United Kingdom	1996	1980–1988	Cohort	179	53.1	69^∧^ (36–92)	59.2	86.0	100.0	NR	NR	63.6 (6–108)
Smith DR	Singapore	2014	NR	Cohort	119	56.3	63.3 (NR)	48.7	NR	100.0	20.2	17.6	28 (NR)
Toon CW	Australia	1996	2004–2009	Cohort	1421	47.9	74^∧^ (17–100)	37.4	38.9	NR	NR	NR	<84 (NR)


*Cohort, Cohort studies; ^∧^Median age; ^∗^mean follow–up time; NR, not reported.*

**Table 2:** The c-Myc information and results of the included studies.

Author	Proportion of positive c-Myc	Test sample	Test content	Test method	Analytic method	Outcome	HR	95% CI
Bhatavdekar JM	64.6	Tissue	Protein	IHC	Uni	OS	3.60	(1.05–12.39)
Böckelman C	28.0	Tissue	Protein	IHC	Uni	OS	0.51	(0.28–0.92)
Erisman MD	68.4	Tissue	RNA	Northern blot	Multi	OS	2.22	(0.68–7.29)
Kakisako K	60.0	Tissue	mRNA	RT-PCR	Uni	DFS^∗^	5.81	(1.02–32.96)
Lee KS	82.8	Tissue	DNA	IHC	Multi	OS	2.35	(1.45–3.80)
Rowley S	73.7	Tissue	DNA	Flow cytometric	Uni	OS	1.21	(0.40–3.67)
Smith DR	60.5	Tissue	RNA	Northern blot	Uni	OS	0.43	(0.20–0.90)
Toon CW	69.0	Tissue	Protein	IHC	Multi	OS	0.91	(0.69–1.20)


*DFS, disease-free survival; HR, hazard ratio; IHC, Immunohistochemistry; Multi, Multivariate analytic method; OS, overall survival; Proportion of positive c-Myc, Proportion of patients with positive c-Myc; RT-PCR, reverse transcription PCR; Uni, Univariate analytic method. ^∗^which was not included for analysis (not OS).*

### Meta-Analysis About OS of c-Myc in CRC Patients

**FIGURE 2:**
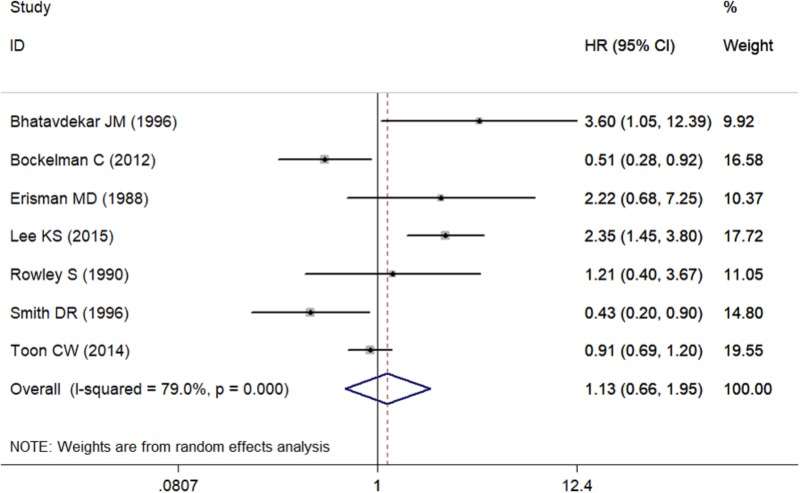
The association between c-Myc and overall survival in seven studies.

Seven studies including 2,712 CRC patients were involved (Table 2). The prognostic roles of c-Myc in CRC were summarized in Figure 2. Inconsistent HRs were observed among studies, suggesting either favorable or poor prognostic roles of c-Myc in CRC. A random-effects model was executed to obtain an unadjusted pooled HR of 1.13 (95% CI: 0.66–1.96, *I*^2^ = 79.0%, *P* < 0.001).

### Subgroup Analysis

**FIGURE 3:**
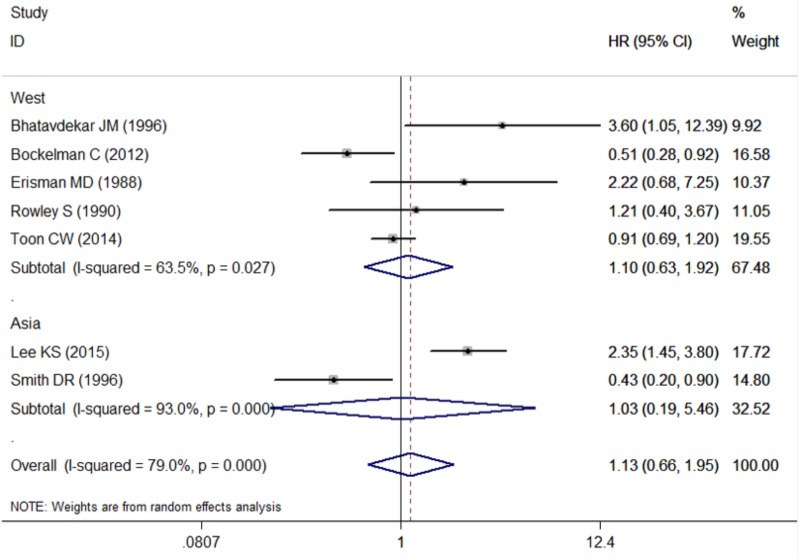
Subgroup analysis for the association between c-Myc and overall survival in the studies from different countries. West, western countries; Asia, Asian countries.

The pooled HR for studies from Western countries was 1.10 (95% CI: 0.63–1.92; *I*^2^ = 63.5%, *P* = 0.027, Figure 3 and Table 3). For studies from Asian countries, the pooled HR was 1.03 (95% CI: 0.19–5.46; *I*^2^ = 93.0%, *P* < 0.001, Figure 3 and Table 3).

**Table 3:** The results of the meta-analysis (OS).

	Number of studies	Patients	HR (95 % CI)	Heterogeneity (*I*^2^, *P*)
**All**	7	2,712	1.26 (0.74–2.17)	78.4%, <0.001
**Country**				
Western	5	2,226	1.10 (0.63–1.92)	63.5%, 0.027
Asian	2	486	1.03 (0.19–5.46)	93.0%, 0.001
**Analytic methods**				
Multivariate analysis	3	2,145	1.57 (0.73–3.39)	83.8%, 0.002
Univariate analysis	4	767	0.86 (0.38–1.94)	71.3%, 0.015
**Test content**				
Protein	3	2,009	0.97 (0.47–1.99)	76.1%, 0.015
RNA	2	157	0.91 (0.18–4.56)	81.3%, 0.021
DNA	2	546	2.05 (1.22–3.46)	13.3%, 0.283


**FIGURE 4:**
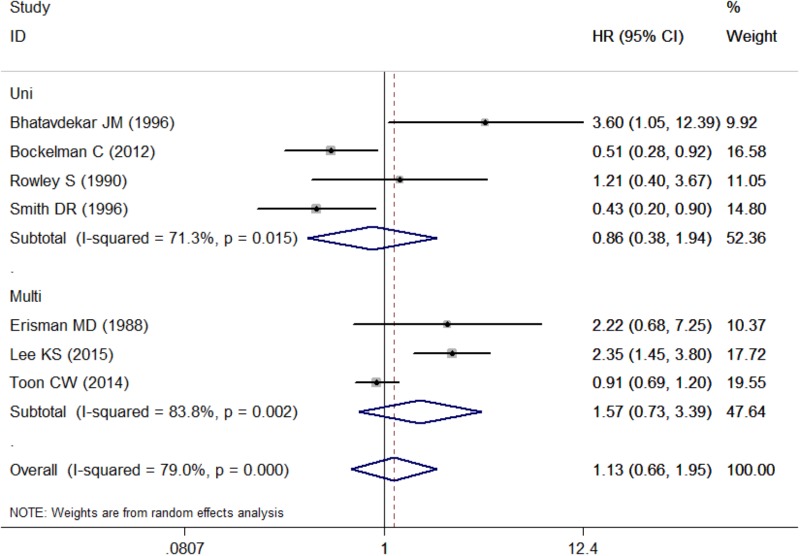
Subgroup analysis for the association between c-Myc and overall survival in the studies using different analytic methods. Uni, univariate analysis; Multi, multivariate analysis.

Pooled HR was 1.57 (95% CI: 0.73–3.39) by combining three studies that provided multivariate analysis (*P* = 0.002, *I*^2^ = 83.8%, Figure 4 and Table 3). In addition, the pooled HR from four studies providing univariate analysis was 0.86 (95% CI: 0.38–1.94) based on the result of random-effect model (*P* = 0.015, *I*^2^ = 71.3%, Figure 4 and Table 3).

**FIGURE 5:**
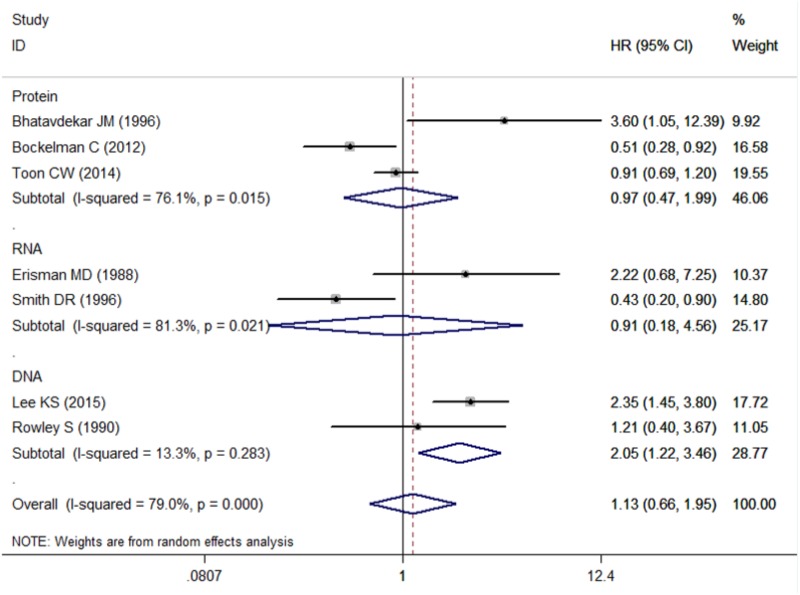
Subgroup analysis for the association between c-Myc and overall survival in the studies using different test content (including Protein, DNA, RNA).

Three studies examined protein level of c-Myc, two studies examined RNA level, while two studies examined DNA level. Pooled HR was 0.97 (95% CI: 0.47–1.99, Figure 5 and Table 3) for protein level of c-Myc, and 0.91 (95% CI: 0.18–4.56) for RNA level. HR from three studies that examined DNA level was 2.05 (95% CI: 1.22–3.46).

### Analysis of Heterogeneity

**FIGURE 6:**
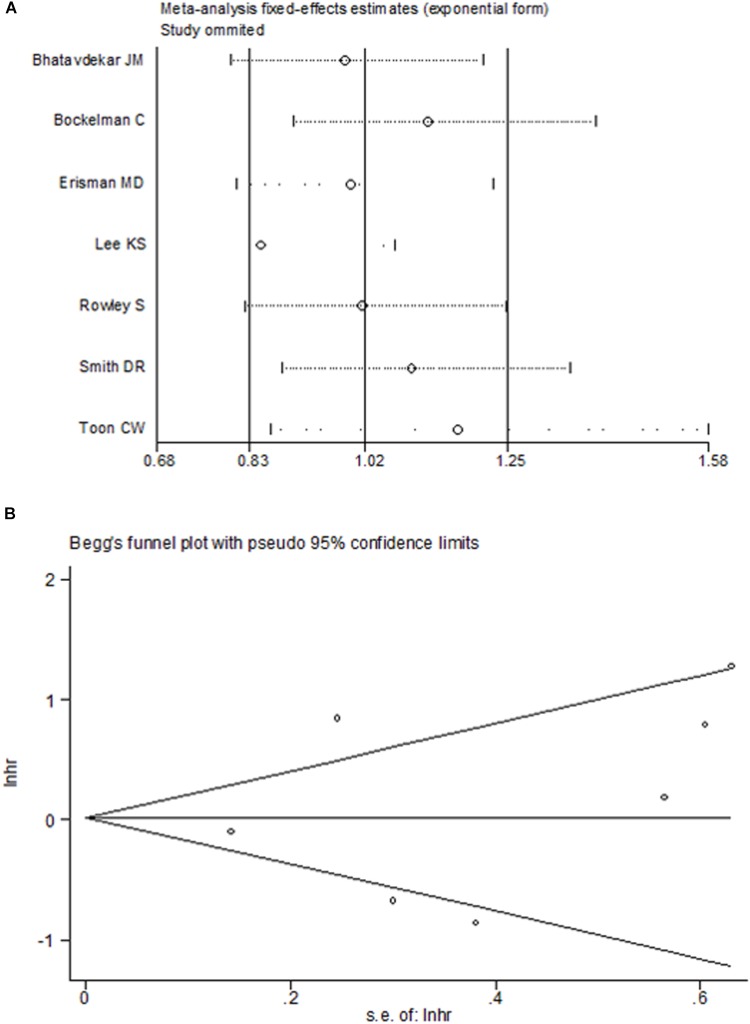
Sensitive analysis **(A)** and Begg’s funnel plot **(B)** for the assessment of included studies in overall survival.

There was significant heterogeneity for OS among seven studies (Figure 2). Meta-regression was performed. The variable “Test content” was related with the heterogeneity of the HRs (Table 4). Sensitivity analysis (Figure 6A) and funnel plot (Figure 6B) were carried out to evaluate the influence of potential publication bias. We did not observe significantly publication bias from egger’s test (*P* = 0.368). However, the shape of the funnel plot indicated some studies were out of the reference line (Figure 6B). Each study in sensitivity analysis was successively removed to evaluate the effect of individual study on the pooled HR (Figure 6A). The results showed that the studies conducted by Bockleman et al. (2012); Toon et al. (2014) were out of the reference line, which demonstrated that there might be publication bias for OS.

**Table 4:** The results of Meta-regression.

	Coef.	SE	*t*-value	*P*	95% CI
Country	0.536	0.434	1.23	0.217	(-0.315–1.387)
Proportion of c-Myc	0.012	0.014	0.83	0.407	(-0.016–0.039)
Test content	0.528	0.264	2.00	0.045	(0.012–1.045)
Analytic method	0.273	0.427	0.64	0.522	(-0.564–1.111)


*Coef, coefficient; SE, standard error.*

## Discussion

This study is the first meta-analysis to examine the association between c-Myc and CRC prognosis. We found that c-Myc was not significantly associated with CRC prognosis.

c-Myc participates in cell proliferation, differentiation, metabolism, survival, and apoptosis by regulating human genes (Guo et al., 2016; Su et al., 2016; Subramaniam et al., 2016). The c-Myc gene can promote tumourigenesis in many types of cancers (Aprelikova et al., 2016; Richart et al., 2016) and plays an important role in the progression of CRC (Smith and Goh, 1996; Kriegl et al., 2012).

Several studies have reported c-Myc status in many cancers, including prostate cancer (Zeng et al., 2015), breast cancer (Elster et al., 2016), and CRC (Lee K.S. et al., 2016). Some cancers with c-Myc overexpression, including oesophageal squamous cell carcinoma, gastric carcinoma, and soft tissue leiomyosarcoma, are correlated with poor survival (Ninomiya et al., 1991; Tsiatis et al., 2009; Wang et al., 2011). Likewise, several cancers with c-Myc gene amplification were associated with poor survival (Dimova et al., 2006; Choi et al., 2012; Seo et al., 2014). However, the prognostic value of c-Myc in CRC patients is quite controversial. It was reported that overexpression of either c-Myc mRNA or c-Myc protein in CRC patients was associated with favorable survival (Smith and Goh, 1996; Toon et al., 2014), but these were opposite results to previous studies that showed that high expression of c-Myc in CRC predicted worse survival outcome (Erisman et al., 1988). The association between c-Myc expression and CRC patients’ prognosis remains debatable. Therefore, it is required to further estimate c-Myc expression in CRC to obtain a conclusion regarding its prognostic value. Therefore, a meta-analysis including 2,947 CRC patients was performed. It was demonstrated that the c-Myc was not significantly associated with CRC prognosis in the overall investigated populations.

In subgroup analysis by ethnicity, we did not detect significant association between c-Myc and survival in either Europeans or Asians, indicating that ethnic differences in genetic backgrounds and the lifestyle context do not influence the association between c-Myc and CRC prognosis.

Nevertheless, there were some limitations in our study. First, adjusted confounding factors, including BMI and environmental factors, varied among studies. What was more, the method of therapy greatly affected the survival time of the CRC patients. Although all of the included patients were diagnosed as CRC, the use of specific therapy differed among the included studies. Thus, the confounding effects of different therapies remain unclear. Second, publication bias was observed among the studies, it might be inevitable due to unpublished studies or original data. Third, test content and evaluation criteria of c-Myc varied among studies, possibly giving rise to significant heterogeneity. HR from three studies that examined DNA level was significantly different, while those about RNA level or protein level were not significantly different. Fourth, only eight studies were enrolled in the meta-analysis, and each study included a relatively small sample size.

Overall, the meta-analysis indicates that c-Myc is not associated with CRC prognosis. However, due to the potential limitations, conclusions must be drawn with caution, and additional larger studies, particularly studies with sub-groups for environmental-genetic interactions, should be performed to validate our findings.

## Author Contributions

W-LH and YP wrote the draft of the paper. X-TW extracted the data and helped to modify the manuscript. J-LW and Y-KL extracted the data. T-WL searched the databases. Q-YZ and YH analyzed the data. X-LC conceived the study, searched the databases and modified the manuscript.

## Conflict of Interest Statement

The authors declare that the research was conducted in the absence of any commercial or financial relationships that could be construed as a potential conflict of interest.
