# Supplementary material for: Association Between c-Myc and Colorectal Cancer Prognosis: A Meta-Analysis
Source: Front Physiol. 2018 Nov 13;9:1549. doi: 10.3389/fphys.2018.01549 (PMC6244870; doi:10.3389/fphys.2018.01549)
Supplement: Supplementary file 1 [file Table_1.DOC]

**Appendix 1. The search strategy**

All the searches were performed on March 14th 2016.

Pubmed

1. “colorectal” OR “large intestine” OR “large bowel” OR “colon” OR “colonic” OR “rectal” OR “rectum” [MeSH Terms]

2. “colorectal” OR “large intestine” OR “large bowel” OR “colon” OR “colonic” OR “rectal” OR “rectum” [Title/Abstract]

3. #1 OR #2 (394 691 articles)

4. “cancer” OR “carcinoma” OR “tumor” OR “tumour” OR “neoplasm” OR “cancers” [MeSH Terms]

5. “cancer” OR “carcinoma” OR “tumor” OR “tumour” OR “neoplasm” OR “cancers” [Title/Abstract]

6. #4 OR #5

7. #3 AND #6

8. MYC OR "bHLH transcription factor" OR "Proto-Oncogene c-Myc" OR "c-Myc" [Title/Abstract]

9. Prognosis OR Prognoses OR Prognostic OR predictive OR biomarker OR marker OR Survival OR Survive OR Cox OR Logrank OR Kaplan-Meier [Title/Abstract]

10. #7 AND #8 AND #9

**Embase**

1. "colorectal Neoplasms"/exp OR "[colonic Neoplasms](http://www.ncbi.nlm.nih.gov/mesh?Db=mesh&Term="Colonic Neoplasms "%5BMESH%5D)"/exp OR "[Rectal Neoplasms](http://www.ncbi.nlm.nih.gov/mesh?Db=mesh&Term="Rectal Neoplasms "%5BMESH%5D)"/exp

2. MYC OR "bHLH transcription factor" OR "Proto-Oncogene c-Myc" OR "c-Myc"

3. Prognosis OR Prognoses OR Prognostic OR predictive OR biomarker OR marker OR Survival OR Survive OR Cox OR Logrank OR Kaplan-Meier

4. #1 AND #2 AND #3

5. #4 AND 'human'/de AND ('colon adenocarcinoma'/de OR 'colon cancer'/de OR 'colon carcinogenesis'/de OR 'colon carcinoma'/de OR 'colon tumor'/de OR 'colorectal cancer'/de OR 'colorectal carcinoma'/de OR 'colorectal tumor'/de) AND 'article'/it

**Cochrane**

1. "colorectal Neoplasms" OR "[colonic Neoplasms](http://www.ncbi.nlm.nih.gov/mesh?Db=mesh&Term="Colonic Neoplasms "%5BMESH%5D)" OR "[Rectal Neoplasms](http://www.ncbi.nlm.nih.gov/mesh?Db=mesh&Term="Rectal Neoplasms "%5BMESH%5D)"

2. MYC OR "bHLH transcription factor" OR "Proto-Oncogene c-Myc" OR "c-Myc"

3. Prognosis OR Prognoses OR Prognostic OR predictive OR biomarker OR marker OR Survival OR Survive OR Cox OR Logrank OR Kaplan-Meier

4. #1 AND #2 AND #3
